# Supplementary material for: Descending motor circuitry required for NT-3 mediated locomotor recovery after spinal cord injury in mice
Source: Nat Commun. 2019 Dec 20;10:5815. doi: 10.1038/s41467-019-13854-3 (PMC6925225; doi:10.1038/s41467-019-13854-3)
Supplement: Supplementary file 1 — Supplementary Information [file 41467_2019_13854_MOESM1_ESM.pdf]

Supplementary Information for

Descending Motor Circuitry Required for NT-3 Mediated Locomotor Recovery after Spinal Cord Injury in Mice

Han *et al.*

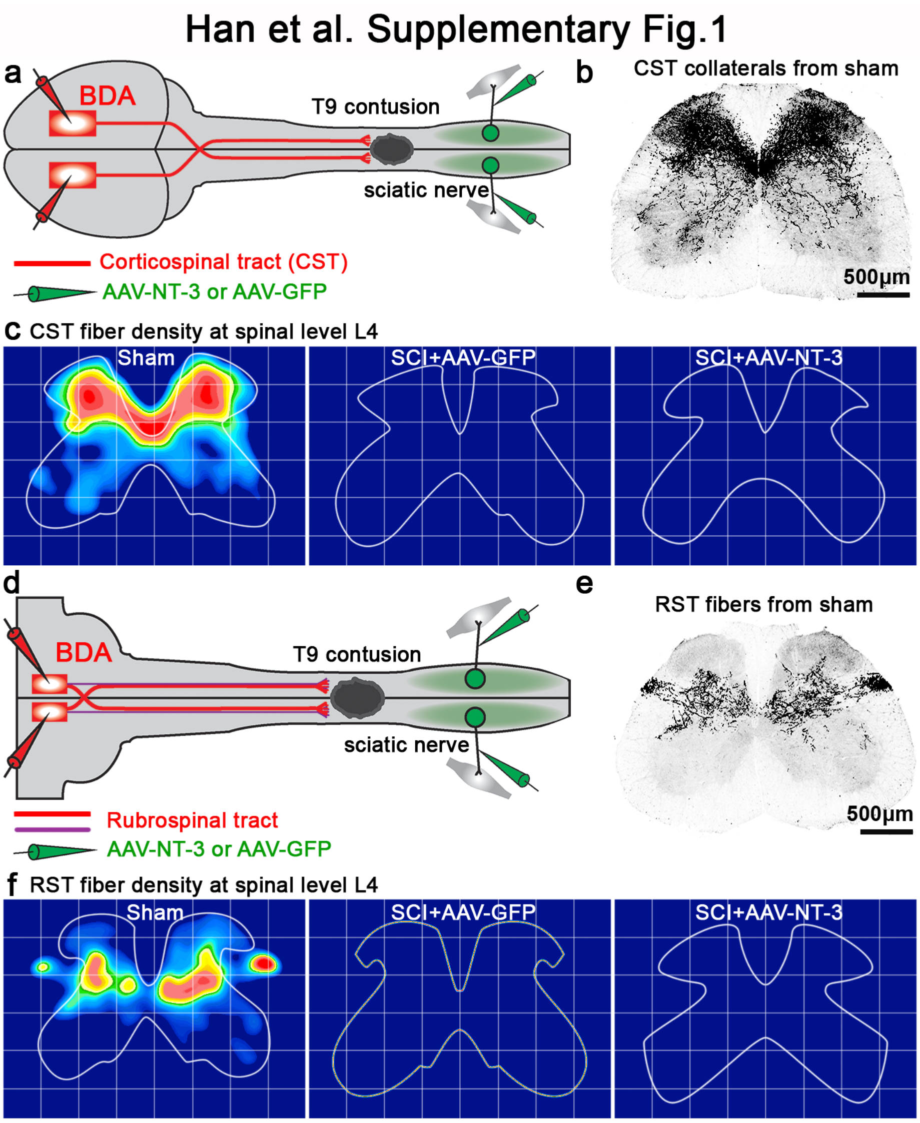

Supplementary Fig. 1. Contusion abolished cortical and rubral projections below the injury site. a Schematic drawing shows the experimental design. A contusive SCI was made at the T9 vertebral level. AAV-NT-3 or AAV-GFP (control) was injected into bilateral sciatic nerves to allow retrograde transport of NT-3 to lumbar MNs. BDA was injected into bilateral sensorimotor cortices to anterogradely label CST axons at 6 wpi. **b** A transverse image shows the distribution of BDA-labeled CST axons in the lumbar spinal cord of a sham control. Scale bar = 500 µm. **c** CST labeling was converted to heatmaps, with red representing the highest number of axon pixels, and blue representing the lowest. The heatmaps show the absence of CST projections in the lumbar spinal cord in contusive mice, regardless of treatments (n = 6-8 mice per group). **d** Schematic drawing showing BDA was bilaterally injected into the red nucleus to anterogradely label the RST axons at 6 wpi. **e** A transverse image shows the RST terminal pattern in the lumbar spinal cord from a sham control. Scale bar = 500 µm. **f** Representative heatmap images of BDA tracing shows the lack of RST projections in contusive mice (n= 6-8 per group). AAV adeno-associated virus, NT-3 neurotrophin-3, GFP green fluorescent protein, BDA Biotinylated dextran amines, CST corticospinal tract, RST rubrospinal tract.

Han et al. Supplementary Fig.2

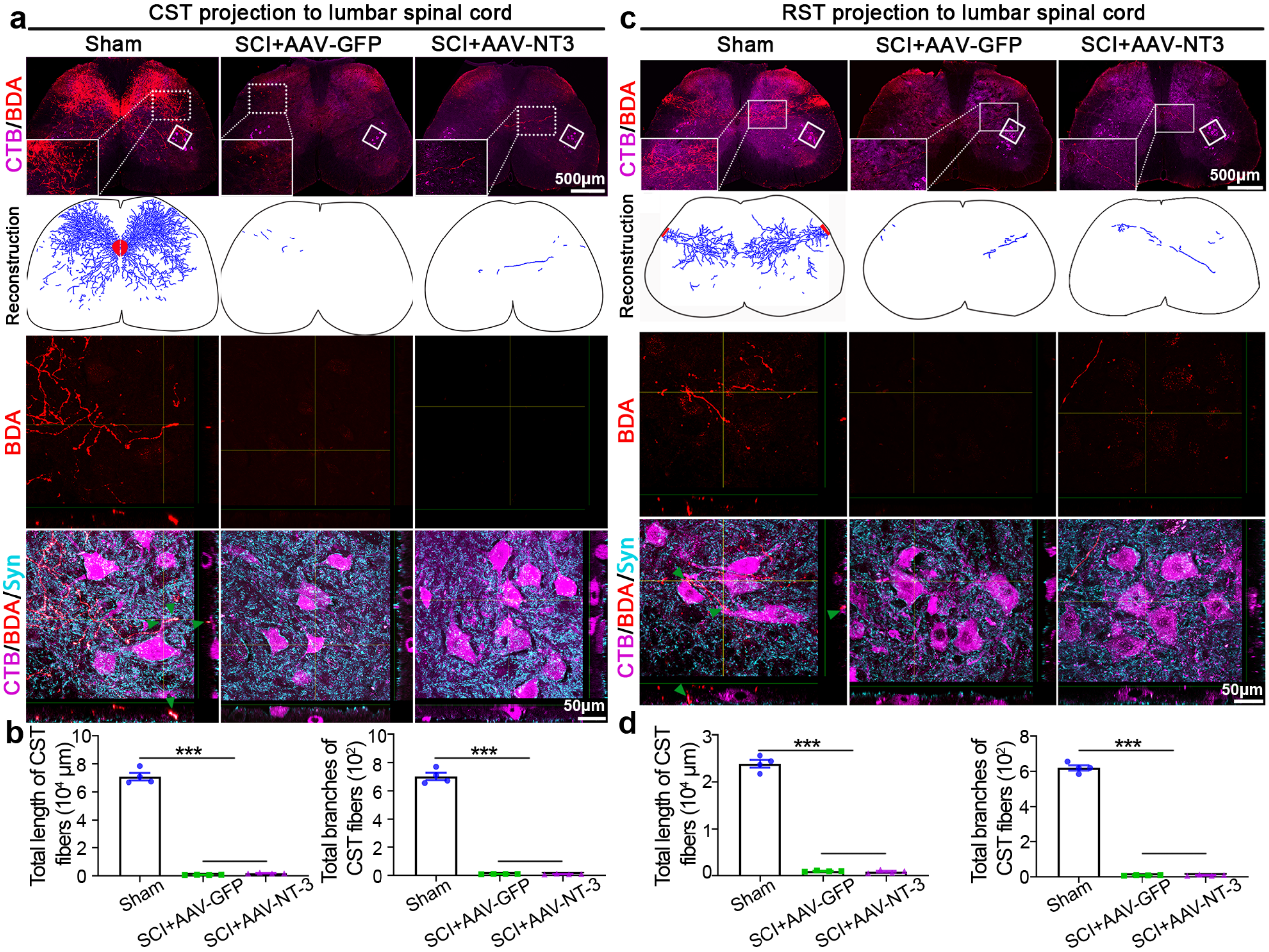

Supplementary Fig. 2. Contusions interrupted corticospino- and rubrospino-MN connections. a Representative images show only few, if any, residual BDA-labelled CST axons project to the lumbar MN pool after a T9 contusion. MNs were retrogradely labeled by CTB. CST collaterals were reconstructed by Imaris. Scale bar = 500 μm. Insets represent the CST distributions in gray matter. The high magnifications of solid boxed regions illustrate CST projections in the lumbar MN pool in sham, SCI+AAV-GFP, and SCI+AAV-NT-3 groups, respectively. Scale bar = 50 μm. **b** Quantitative analysis of CST fiber lengths and segments among the 3 groups. **c** Representative images show BDA-labelled residual RST distribution in the CTB-labeled lumbar MNs. The RST projections were reconstructed by the Imaris. Scale bar = 500 μm Insets represent the RST distributions in gray matter. The high magnifications derived from solid boxed regions illustrate RST projections in the lumbar MNs in sham, SCI+AAV-GFP, and SCI+AAV-NT-3 mice, respectively. Scale bar = 50 μm. **d** Quantitative analysis of the RST fiber lengths and branches among the 3 groups. Dots in bar charts represent the animal numbers in each group. Data are presented as mean ± SEM. n = 4 biologically independent animals per group. ***$P < 0.001$. One-way ANOVA followed by Tukey's post hoc test. MN motoneuron, CTB cholera toxin subunit B, Syn synaptophysin. Source data are provided as a Source Data file.

Han et al. Supplementary Fig.3

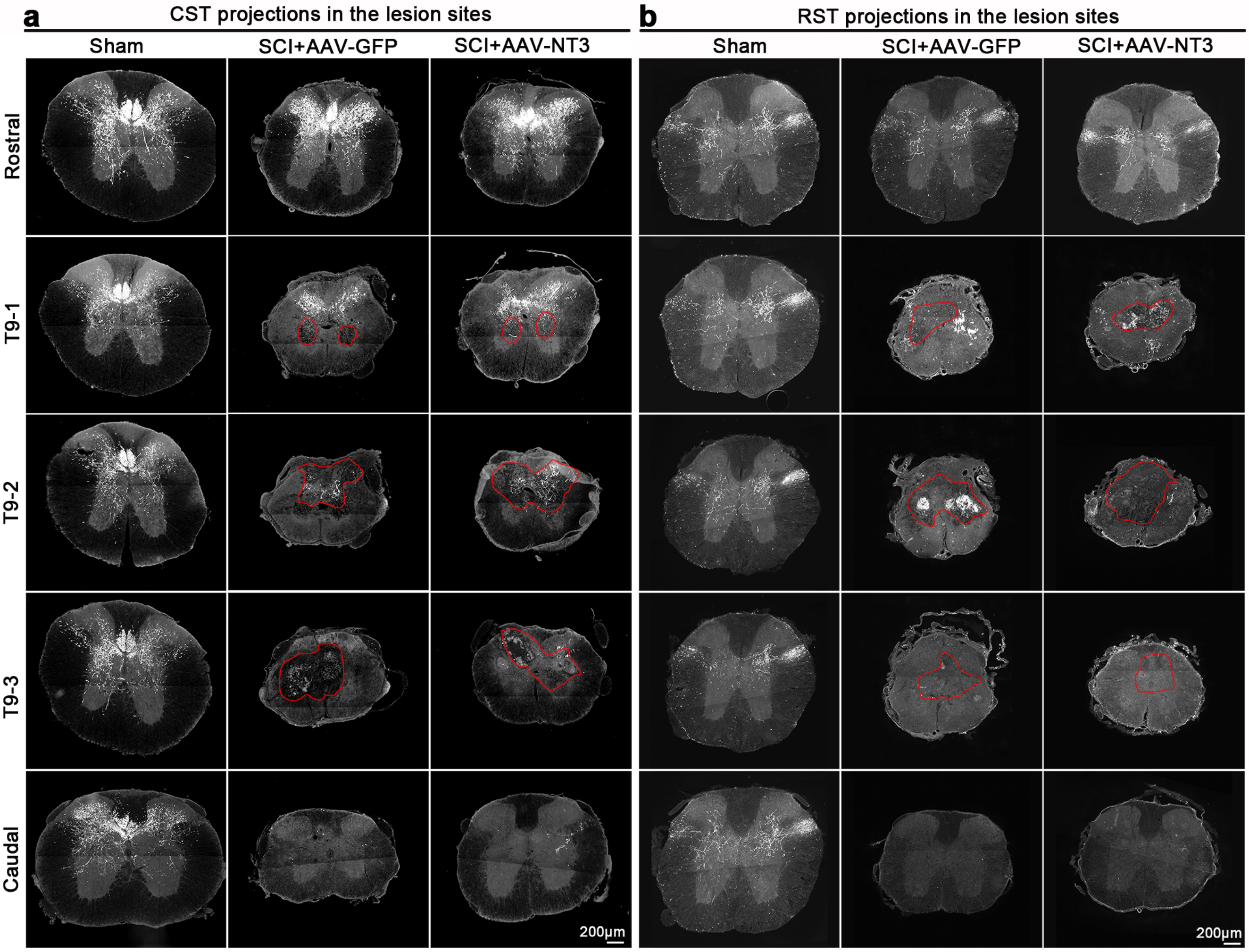

Supplementary Fig. 3. Rostrocaudal distributions of CST and RST after contusion. a A series of representative images at different spinal levels show CST projection in the sham, SCI+AAV-GFP, and SCI+AAV-NT-3 groups. Images are in order of rostral to caudal, sequentially. Red circles indicate the lesion centers. Scale bar = 200 μm. **b** Rostrocaudal distribution of the RST axons after a T9 contusion. A series of representative images from each experimental group show the RST axonal projection rostral, at, and caudal to the level of injury. Red outlines indicate the lesion centers. Scale bar = 200 μm.

Han et al. Supplementary Fig.4

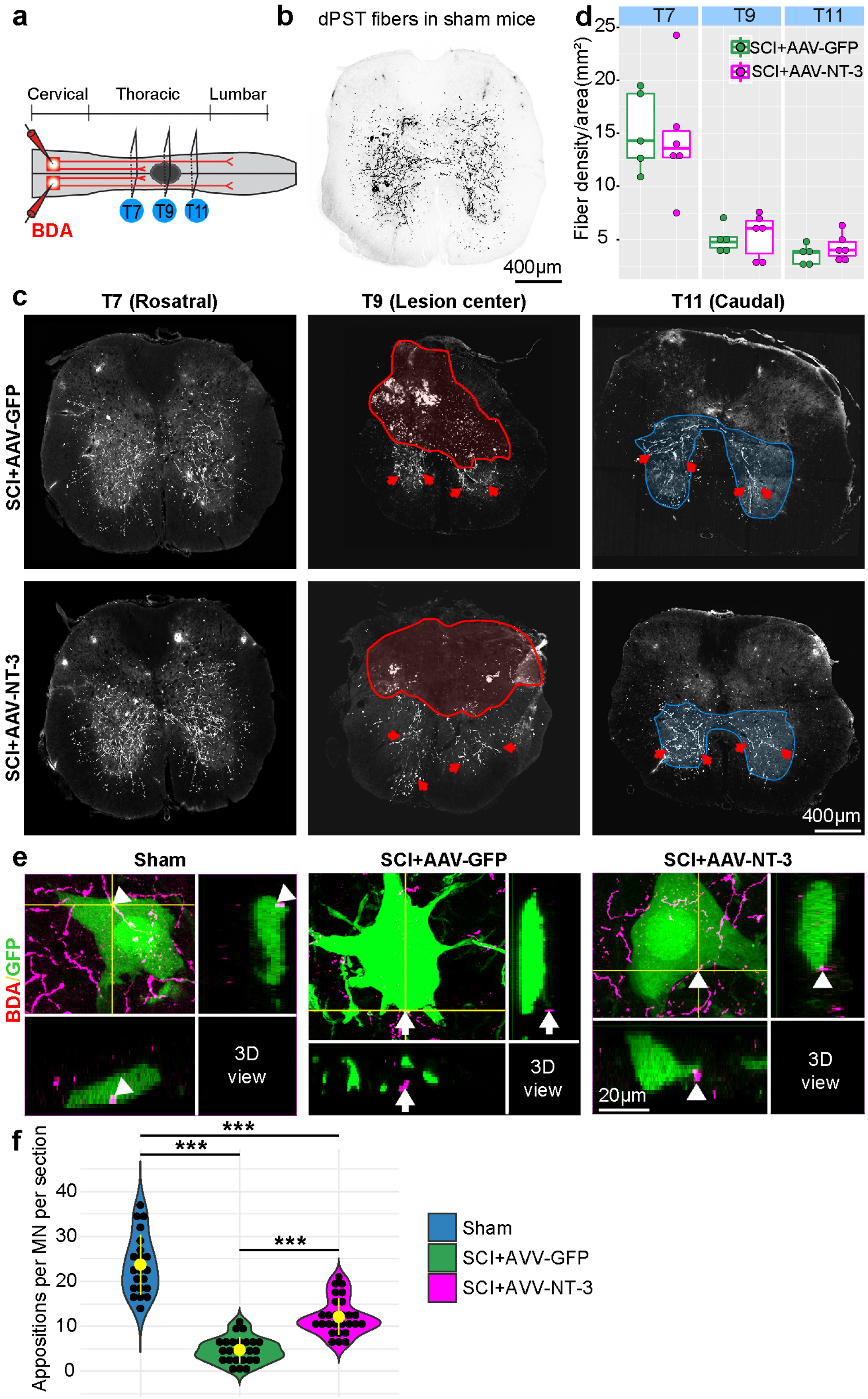

Supplementary Fig. 4. Rostrocaudal distribution of cervical dPST axons after contusion. a The schematic shows BDA injection into the cervical spinal cord (C5-C6) to label the cervical dPST. Samples were taken from levels rostral (T7), at (T9) and caudal (T11) to the injury. **b** Representative images show BDA-labeled dPST axons at T7 in a sham mouse. Scale bar = 400 μm. **c** A series of representative images show the dPST axonal distribution in the spinal cord at rostral (T7), at (T9), and caudal (T11) to the lesion. Red contours indicate the lesion area. Blue contours indicate dPST distribution below the lesion site. Arrows indicate the spared dPST axons at and below the lesion. Scale bar = 400 μm. **d** Quantitative analysis of the dPST fiber density above, at, and below the lesion site between SCI+AAV-GFP and SCI+AAV-NT-3 groups. Data are presented as box plots with center lines indicating medians, boxes representing 25th to 75th percentiles, and whiskers representing data points within 1.5 times the IQR. n = 5-6 biologically independent animals in each group. Unpaired two-tailed Student t-test. **e** Representative images show the BDA-labelled dPST axons innervating lumbar MNs. Arrowheads represent three-dimensional appositions; arrows represent one-dimensional apposition. Scale bar = 20 μm. **f** Violin plots indicate the three-dimensional apposition numbers per MN soma in defined tissue volume. The dots in the violin plots represent triple appositions in each group. The yellow lines indicate mean ± SD. n = 4-6 biologically independent animals per group. ***P < 0.001; One-way ANOVA followed by Tukey's post hoc test. dPST descending propriospinal tract. Source data are provided as a Source Data file.

Han et al. Supplementary Fig.5

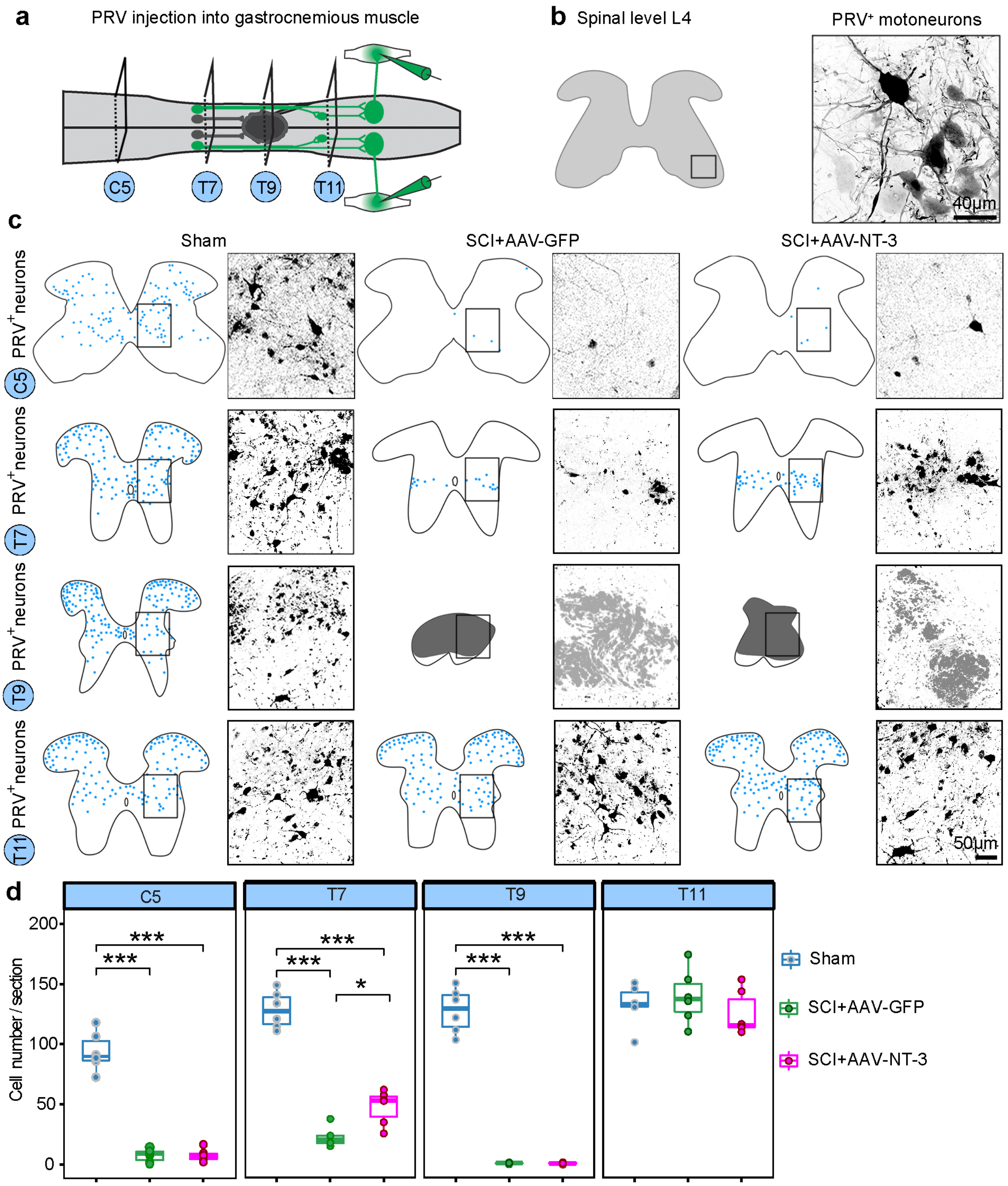

Supplementary Fig. 5. NT-3 increased propriospino-MN connections after contusion. a Diagram shows PRV injected into the gastrocnemius muscles for trans-synaptically labeling of different orders of spinal neurons. **b** PRV labeled (PRV⁺) lumbar MNs in the lumbar ventral horn (boxed area) at 48h after inoculation. Scale bar = 400 μm. **c** Representative reconstructions show the distribution of PRV⁺ dPNs from above (C5 and T7), at (T9), and below (T11) the lesion site in each experimental group at 72 h post-injection. Insets indicate PRV⁺ dPNs in boxed areas of respective plots. Scale bar = 50 μm. **d** Box plots show the number of PRV⁺ dPNs at C5, T7, T9 and T11 segments. Data are presented as box plots with center lines indicating medians, boxes representing 25th to 75th percentiles, and whiskers representing data points within 1.5 times the IQR. n = 5-6 biologically independent animals per group. **P* < 0.05; ****P* < 0.001. One-way ANOVA followed by Tukey's post hoc test. PRV pseudorabies virus, dPNs descending propriospinal neurons. Source data are provided as a Source Data file.

Han et al. Supplementary Fig.6

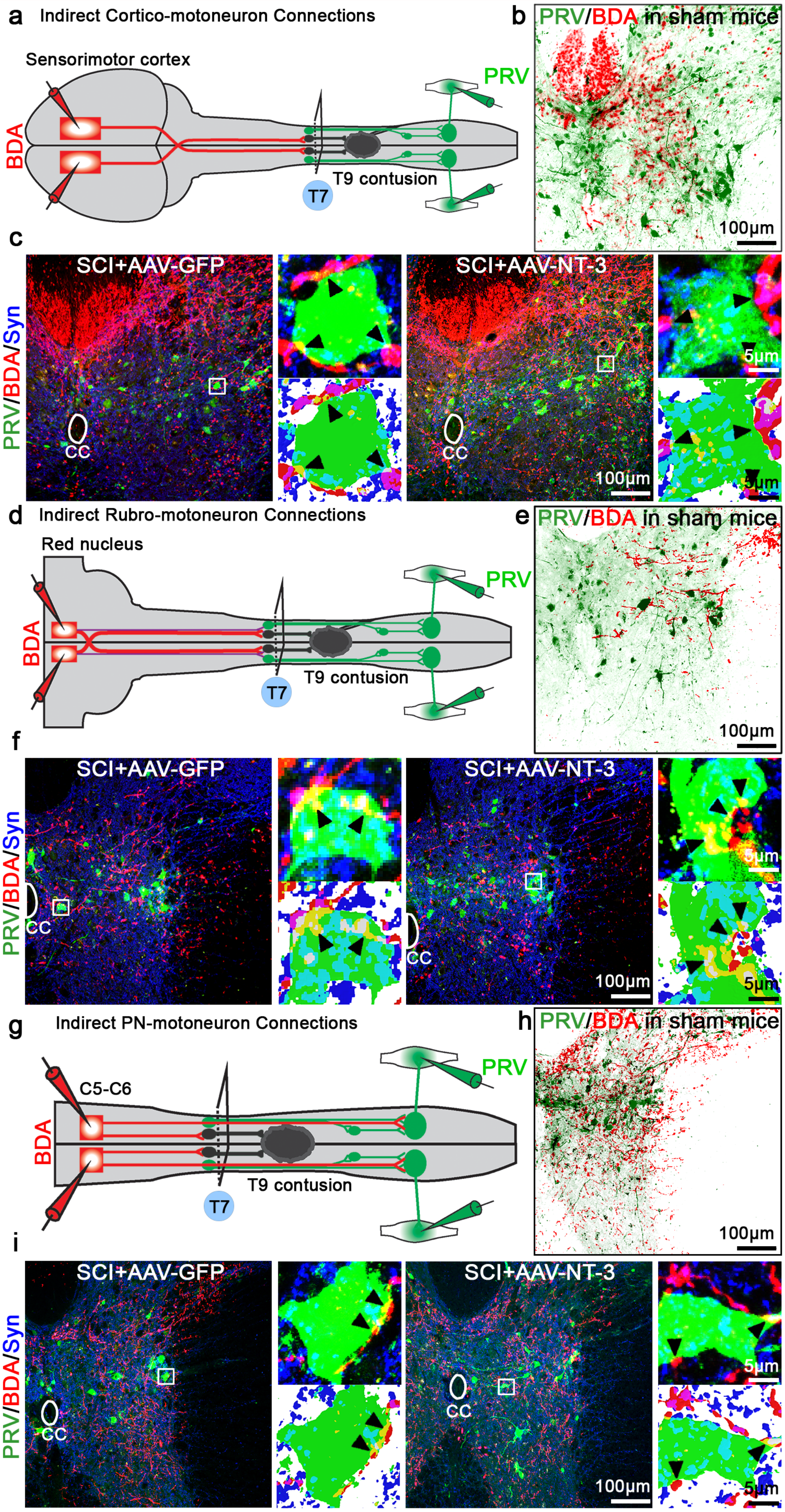

Supplementary Fig. 6. dPNs relayed superspinal and propriospinal commands below injury. a A diagram illustrates the strategy for detecting synaptic connections between BDA-labelled CST axons and PRV-labelled dPNs at the T7 level. **b** Representative image shows BDA-labelled CST project to PRV-labeled dPNs at T7. Scale bar = 100 μm. **c** Representative confocal images show triple labeling of the CST axons (red), dPNs (green) and synaptophysin (SYP, a presynaptic marker, blue). Scale bar, 100 μm. High magnification images from boxed areas show close triple-appositions (upper) and Imaris-reconstructed synaptic-like contacts (lower), which were indicated by arrowheads. Scale bar = 5 μm. CC, central canal. **d** The diagram illustrates the strategy for detecting contacts between BDA-labelled RST axons and PRV-labelled dPNs above the contusion. **e** Representative image shows that the RST axons project to dPNs at T7 in a sham mouse. Scale bar =100 μm. **f** Representative images show triple labeling between the RST axons (red), dPNs (green), and synaptophysin (blue). High magnification images from boxed areas indicate close triple-appositions and Imaris-reconstructed synaptic-like contacts, indicated by arrowheads. Scale bar = 5 μm. **g** The diagram illustrates the strategy for detecting synaptic connections between BDA-labelled cervical dPST axons and PRV-labelled thoracic dPNs rostral to the T9 contusion. **h** Representative image shows the projection of cervical dPST axons on thoracic dPNs at T7 in a sham mouse. Scale bar = 100 μm**. i** Representative images show triple labeling between cervical dPST (red), thoracic dPNs (green), and synaptophysin (blue) at T7 (above the contusion). High magnification images from boxed areas indicate close triple-appositions and Imaris-reconstructed synaptic-like contacts, which were indicated as arrowheads. Scale bar = 5 μm. dPNs descending propriospinal neurons.

Han et al. Supplementary Fig.7

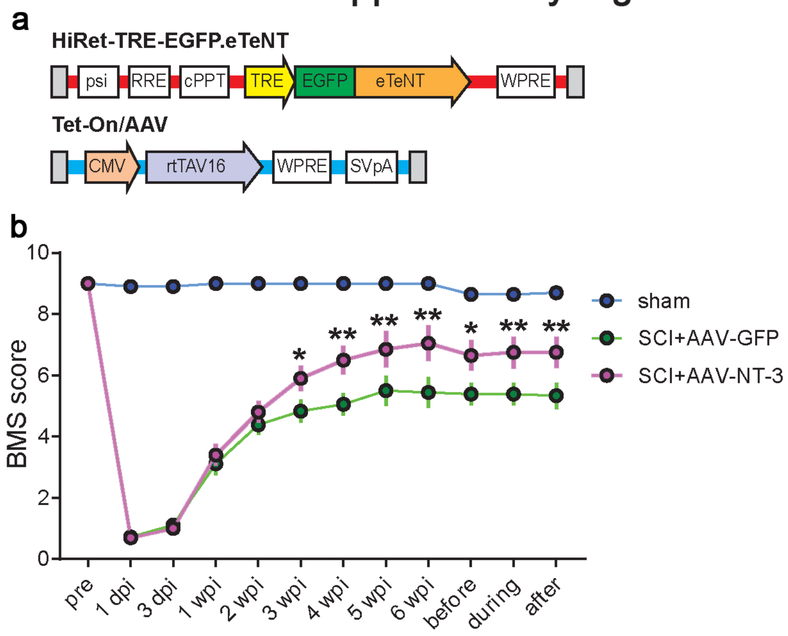

Supplementary Fig. 7 The design of viral vectors and BMS motor behavioral test. a The design of viral vectors. **b** Line plot shows BMS score difference between sham, SCI+AAV-GFP, and SCI+AAV-NT-3 groups, at time points before, during and after Dox administration. Data are presented as mean ± SEM. n = 9-10 biologically independent animals per group. **P* < 0.05, ***P* < 0.01 (SCI+AAV-GFP vs SCI+AAV-NT-3). One-way ANOVA followed by Tukey's post hoc test. cPPT, central polypurine tract; RRE, Rev responsive element; psi, packaging signal; TRE, tetracycline-responsive element, EGFP, enhanced GFP; eTeNT, enhanced tetanus neurotoxin light chain; CMV, cytomegalovirus; rtTAV16, a variant of reverse tetracycline transactivator; WPRE, Woodchuck hepatitis virus post-transcriptional regulatory element; Dox doxycycline. Source data are provided as a Source Data file.

Han et al. Supplementary Fig.8

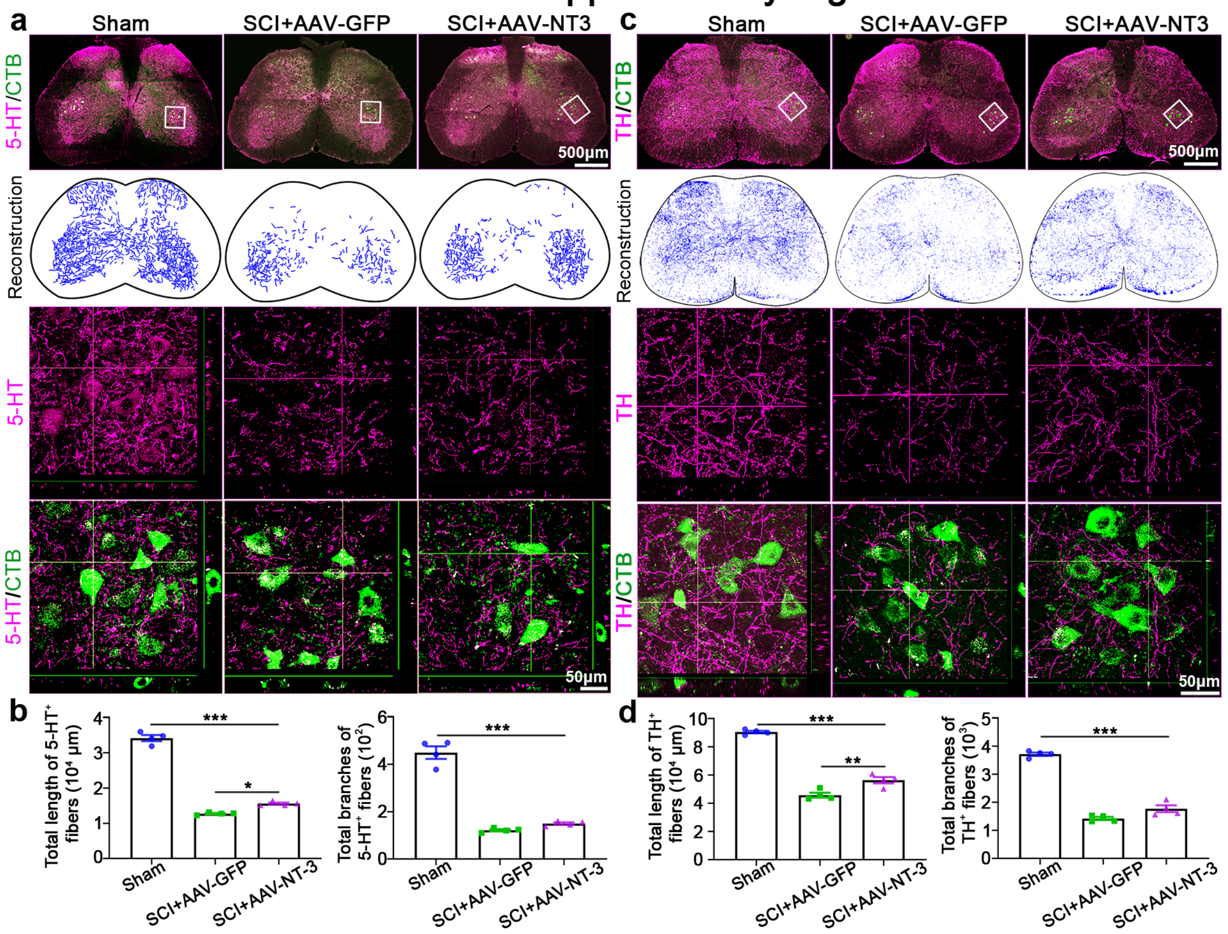

Supplementary Fig. 8. NT-3 increased sprouting of descending serotoninergic and dopaminergic terminals. a Representative images show the residual serotonin immunoreactive (5-HT⁺) fiber distribution in the lumbar spinal cord. 5-HT⁺ axon distributions were reconstructed by Imaris. Scale bar = 500 μm. High magnifications of boxed areas illustrate 5-HT⁺ projections in the lumbar MN pools from sham, SCI+AAV-GFP and SCI+AAV-NT-3 groups, respectively. Scale bar = 50 μm. **b** Quantitative analysis of 5-HT⁺ fiber lengths and branches among the three groups. **c** Representative images show the residual dopaminergic immunoreactive (TH⁺) fiber distribution in the lumbar spinal cord. TH⁺ axon distributions were reconstructed by Imaris. Scale bar = 500 μm. High magnifications of boxed areas illustrate TH⁺ axonal projections in the lumbar MN pools from the sham, SCI+AAV-GFP and SCI+AAV-NT-3 groups, respectively. Scale bar = 50 μm. **d** Quantitative analysis of TH immunoreactive fiber lengths and branches Dots in bar charts represent the animal numbers in each group. Data are presented as mean ± SEM. n = 4 biologically independent animals per group. *P < 0.05, **P < 0.01. One-way ANOVA followed by Tukey's post hoc test. 5-HT 5-hydroxytryptamine/serotonin, TH tyrosine hydroxylase. Source data are provided as a Source Data file.

Han et al. Supplementary Fig.9

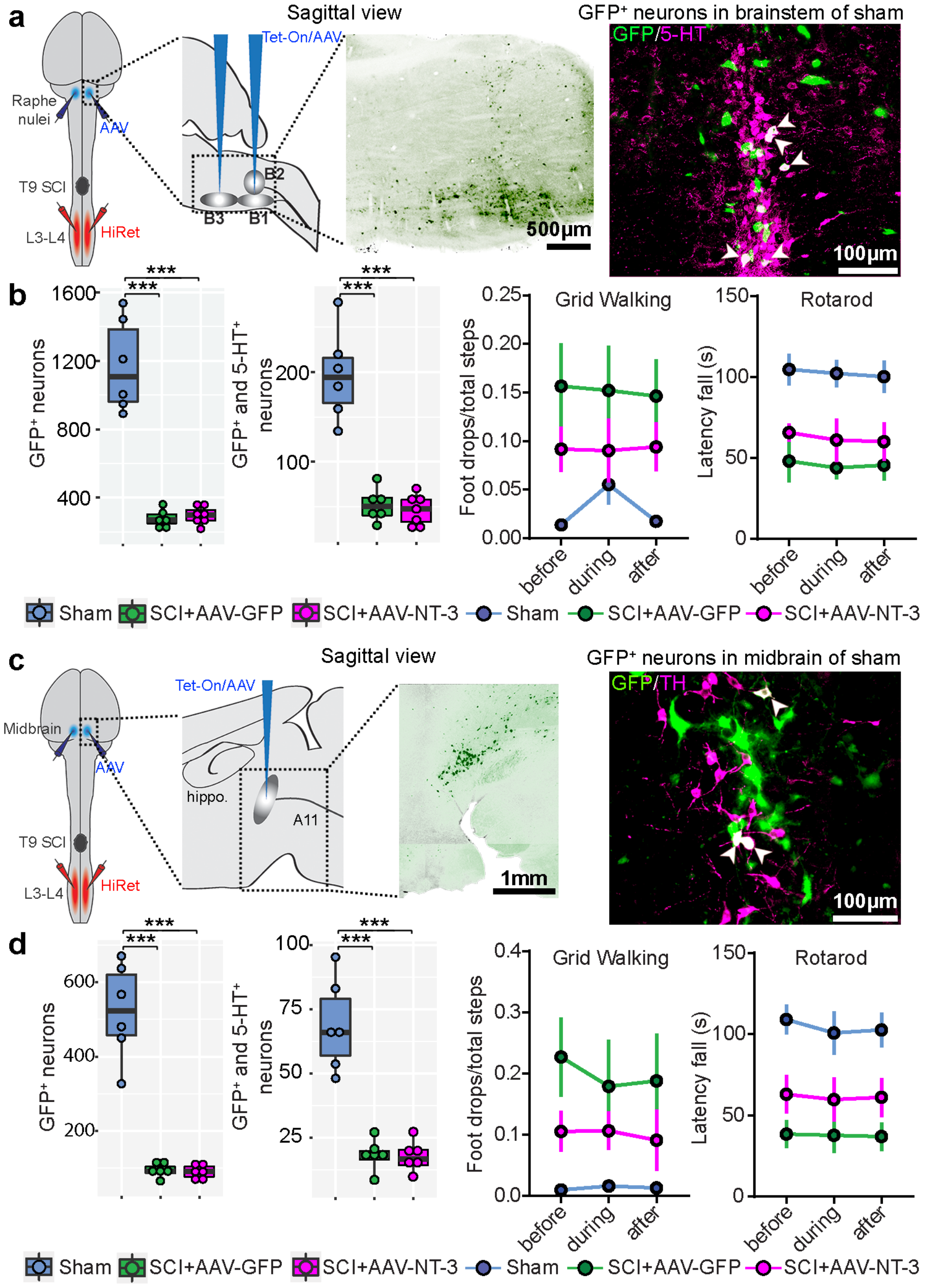

Supplementary Fig. 9. Silencing monoaminergic pathways failed to block NT-3-mediated motor recovery. a Scheme illustrates the selective silencing of the spared serotoninergic pathway in contusive mice, with HiRet-TRE-EGFP-eTeNT injected into the L2-L4 spinal cord, and Tet-On/AAV injected into the raphe nucleus. Insets represent the sagittal view of Tet-On/AAV injections into B1-B3 raphe nuclei. Scale bar = 500 μm. A representative image illustrates the distribution of 5-HT$^+$ and GFP positive (GFP$^+$) neurons in the raphe nucleus. Scale bar = 100 μm. **b** Box plots demonstrate the total number of GFP$^+$ neurons and GFP$^+$ and 5-HT$^+$ double-labeled neurons in the brainstem from each experimental group. Data are presented as box plots with center lines indicating medians, boxes representing 25th to 75th percentiles, and whiskers representing data points within 1.5 times the IQR. n = 6-7 biologically independent animals per group. ***$P < 0.001$. One-way ANOVA followed by Tukey's post hoc test. Line plots indicate changes in grid walking and rotarod tests before, during and after Dox administration. Data are presented as mean ± SEM. n = 6-7 biologically independent animals per group. Two-way ANOVA followed by Tukey's post hoc test. **c** Scheme illustrates the selective silencing of the spared dopaminergic pathway in contusive mice, with HiRet-TRE-EGFP-eTeNT injected into the L2-L4 spinal cord, and Tet-On/AAV injected into the midbrain A11 region. Insets represent the sagittal view of Tet-On/AAV injections into A11 neuron cluster. Scale bar = 1mm. Representative image shows the distribution of TH$^+$ and GFP$^+$ neurons in the A11 cluster region. Scale bar = 100 μm. **d** Bar plots report the total number of GFP$^+$ neurons and GFP$^+$ and TH$^+$ double-labeled neurons in the midbrain from each experimental group. Data are presented as box plots with center lines indicating medians, boxes representing 25th to 75th percentiles, and whiskers representing data points within 1.5 times the IQR. n = 6 biologically independent animals per group. ***$P < 0.001$. One-way ANOVA followed by Tukey's post hoc test. Line plots indicate alterations in grid walking and rotarod before, during and after Dox administration. Data are presented as mean ± SEM. n = 6 biologically independent animals per group. Two-way ANOVA followed by Tukey's post hoc test. Source data are provided as a Source Data file.
